# Comparative transcriptome profiling of heat stress response of the mangrove crab *Scylla serrata* across sites of varying climate profiles

**DOI:** 10.1186/s12864-021-07891-w

**Published:** 2021-07-29

**Authors:** Anish M.S. Shrestha, Crissa Ann I. Lilagan, Joyce Emlyn B. Guiao, Maria Rowena R. Romana-Eguia, Ma. Carmen Ablan Lagman

**Affiliations:** 1 Bioinformatics Lab, Advanced Research Institute for Informatics, Computing, and Networking (AdRIC), De La Salle University, Manila, Philippines; 2 Software Technology Department, College of Computer Studies, De La Salle University, Manila, Philippines; 3 Practical Genomics Laboratory, Center for Natural Science and Environment Research, De La Salle University, Manila, Philippines; 4 Department of Biological Sciences, College of Science, University of Santo Tomas, Manila, Philippines; 5 Mathematics and Statistics Department, College of Science, De La Salle University, Manila, Philippines; 6 Aquaculture Department, Southeast Asian Fisheries Development Center, Binangoan, 1940 Rizal, Philippines; 7 Biology Department, College of Science, De La Salle University, Manila, Philippines

**Keywords:** Mud crab, Mangrove crab, *Scylla*, RNA-seq, Transcriptome assembly, Heat stress

## Abstract

**Background:**

The fishery and aquaculture of the widely distributed mangrove crab *Scylla serrata* is a steadily growing, high-value, global industry. Climate change poses a risk to this industry as temperature elevations are expected to threaten the mangrove crab habitat and the supply of mangrove crab juveniles from the wild. It is therefore important to understand the genomic and molecular basis of how mangrove crab populations from sites with different climate profiles respond to heat stress. Towards this, we performed RNA-seq on the gill tissue of *S. serrata* individuals sampled from 3 sites (Cagayan, Bicol, and Bataan) in the Philippines, under normal and heat-stressed conditions. To compare the transcriptome expression profiles, we designed a 2-factor generalized linear model containing interaction terms, which allowed us to simultaneously analyze within-site response to heat-stress and across-site differences in the response.

**Results:**

We present the first ever transcriptome assembly of *S. serrata* obtained from a data set containing 66 Gbases of cleaned RNA-seq reads. With lowly-expressed and short contigs excluded, the assembly contains roughly 17,000 genes with an N50 length of 2,366 bp. Our assembly contains many almost full-length transcripts – 5229 shrimp and 3049 fruit fly proteins have alignments that cover >80% of their sequence lengths to a contig. Differential expression analysis found population-specific differences in heat-stress response. Within-site analysis of heat-stress response showed 177, 755, and 221 differentially expressed (DE) genes in the Cagayan, Bataan, and Bicol group, respectively. Across-site analysis showed that between Cagayan and Bataan, there were 389 genes associated with 48 signaling and stress-response pathways, for which there was an effect of site in the response to heat; and between Cagayan and Bicol, there were 101 such genes affecting 8 pathways.

**Conclusion:**

In light of previous work on climate profiling and on population genetics of marine species in the Philippines, our findings suggest that the variation in thermal response among populations might be derived from acclimatory plasticity due to pre-exposure to extreme temperature variations or from population structure shaped by connectivity which leads to adaptive genetic differences among populations.

**Supplementary Information:**

The online version contains supplementary material available at (10.1186/s12864-021-07891-w).

## Background

*Scylla serrata* is a commercially important, portunid mangrove crab. Of the four species that currently constitute the genus *Scylla* [1, 2], *S. serrata* is the most widely distributed – occurring throughout coastal tropical and subtropical Indo-Pacific [3, 4]. It is also the preferred species for farming due to its fast growth rate and large size [5]. The fishery and aquaculture of mangrove crabs is a steadily growing, high-value, global industry [4]. In the Philippines alone, its production increased from 17,000 metric tons in 2013 to almost 22,000 metric tons in 2018 [6, 7].

Fluctuations in climate, particularly that of elevation in temperature, pose a threat to the mangrove crab industry. Rising sea surface temperatures are expected to threaten the environment in which the mangrove crabs thrive and the supply of mangrove crab juveniles from the wild. Adverse effects of high temperature to the development and survival of ectothermic crustaceans [8–11], including *S. serrata* [12] are well documented. These studies however, have not looked into the biogeographic region as a factor in stress response. Investigating differences in temperature stress response of crabs from different sites may help to understand anecdotal reports that farmers prefer to source juveniles from certain areas and hatchery operators choose to breed crabs from specific locations. In addition, a high degree of intra- and inter-species genetic diversity among mangrove crabs from different regions [13–16] have been reported. Observed population genetic structures were shown to be associated with reproductive and ecological processes including surface temperature gradients in the case of *S. olivacea* [15]. Assuming a similar relationship applies to *S. serrata*, will mangrove crabs from sites with higher temperature fluctuations have different responses to temperature stress than those with lower fluctuations? One approach to this question is to look into the differences in the molecular mechanisms by which mangrove crab populations from sites with different climate profiles respond to temperature stress.

Past studies on stress response in mangrove crabs have been limited to using qPCR to evaluate the expression level variations of a limited number of genes, such as the heat-shock proteins [17]. Similar studies in other crustaceans such as amphipods, clams, and shrimps have also been reported [18–20]. However, the qPCR approach is unable to detect responses that are multi-genic in nature or that involve new genes not yet known to be associated with stress response.

**Fig. 1:**
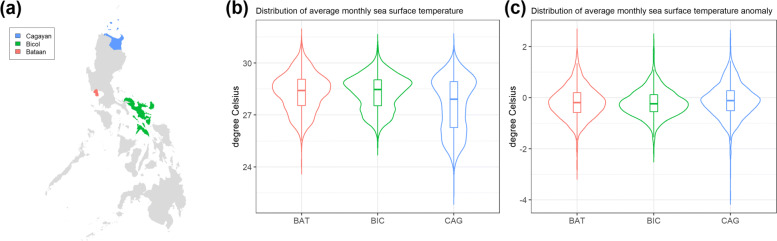
**(a)** The 3 regions in the Philippines, Bataan (BAT), Bicol (BIC), and Cagayan (CAG), from where the *S. serrata* individuals were sampled. The map was produced using the R package sp [21] based on data from the Data of Global Administrative Areas, GADM version 3.6. [22]. The data are freely available for academic use and other non-commercial use. **(b)** Distribution of monthly average temperature between 1900 – 2019 and **(c)** Distribution of monthly average sea-surface temperature anomaly. Sea-surface temperature data was obtained from ERSST version 5 [23]

In this study, we used RNA-seq to compare the transcriptome expression profiles of the gill tissue of 29 *S. serrata* samples from 3 different populations in the Philippines, under normal and heat-stressed conditions. Cagayan, Bicol, and Bataan are located in North, South, and Central Luzon, respectively. Cagayan and Bicol lie on the eastern coast facing the Philippine Sea, while Bataan faces the South China Sea to the west of the Philippines (Fig. 1a). They are major sites where crabs are caught, farmed, and fattened. The three sites fall in different climate zones and witness different weather profiles [24]. Of particular relevance to this study is sea-surface temperature. We show in Fig. 1, the distribution of historical sea-surface temperatures. Cagayan experiences a more extreme weather profile, with both a wider range of annual sea-surface temperature (Fig. 1b) and a wider range of temperature anomalies (Fig. 1c). Bataan, which is latitudinally close to Bicol, witnesses similar average sea-surface temperature as Bicol, but with a slightly larger variance. Of the three, Bicol witnesses the least amount of variation.

To compare the transcriptome expression profiles of the 6 groups (3 sites ×2 conditions), we designed a 2-factor generalized linear model containing interaction terms, which allowed us to simultaneously analyze within-site response to heat-stress and across-site differences in the response. In the within-site analysis, we identified 177, 755, and 221 differentially expressed genes in the Cagayan, Bataan, and Bicol group, respectively. In the across-site analysis between Cagayan and Bataan, we identified 389 genes associated with 48 signalling and stress-response pathways, for which there was an effect of site in the heat response. Between Cagayan and Bicol, there were 101 such genes affecting 8 pathway. Through homology search and gene ontology analysis, we discovered that many of these genes are known to be associated with stress response pathways. Well-annotated reference transcriptome or genome resources are unavailable for mangrove crabs and scant for crustaceans in general [25]. As part of the RNA-seq data analysis procedure, we computed the first ever transcriptome assembly of any tissue of *S. serrata* containing roughly 17,000 genes and N50 length of 2,366 bp. The assembly contains several thousands of transcripts that have long, high-confident alignments against sequences from the fruit fly proteome, UniProt Swiss-Prot, and the shrimp proteome.

## Results

### Study design and data

Our raw data consists of a total of 725 million 100 bp-long paired-end RNA-seq reads. These were obtained from the gill tissue of 29 adult *S. serrata* individuals that came from 6 groups – a combination of 3 sites (Cagayan, Bicol, and Bataan) and 2 treatments (Control and Heat-stressed). The Bataan heat-stressed group had 4 individuals due to 1 sample having low RNA quality, all others had 5. Individuals were sequenced at an average depth of 25 million reads. After removing low-quality read segments and potential rRNA-derived reads, there were a total of 663 million reads remaining. Prior to gene expression analysis, we conducted quality check of the expression data(details in the Supplementary Information), which led us to omit 1 sample each from the two Cagayan groups and the Bicol control group due to poor within-group expression correlation.

### Transcriptome assembly

From the cleaned reads, we obtained a de-novo transcriptome assembly of size 402 million bases containing 505,246 contigs grouped into 349,812 ‘genes’. The N50 statistic was 1,467 bp when accounting for all contigs, and 678 bp when selecting only one longest isoform per gene. Applying CD-HIT to cluster sequences with 95% similarity, reduced the number of transcripts to 435,969 and genes to 345,516.

**Fig. 2:**
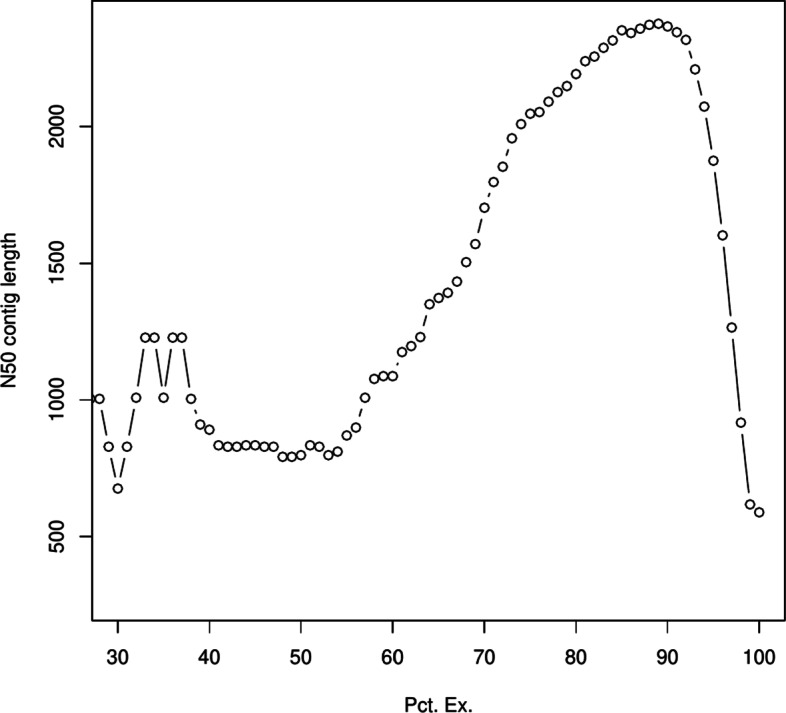
ExN50, which is the N50 length when considering only the top expressing genes that account for *x* percentage of expression, shown here for different values of *x*

An overwhelming majority of the estimated genes were lowly-expressed and have short transcripts. This can be seen by considering the subset *S*_*x*_ of genes containing only the top highly expressed ones which account for *x* percent of the total expression – where gene expression is the sum of TMM value [26] across all isoforms and all samples. For *x*=90,*S*_*x*_ contains 17,162 out of the initial 349,812 genes. Further, as shown in Fig. 2, the ExN50 statistic – which is the N50 value for *S*_*x*_ with the length of a gene defined as the average length of its isoforms weighed by their expression – peaks at N50 length of 2,366 bp around *x*=90.

### Assembly quality assessment

We evaluated the quality and contiguity of the assembly using several metrics.

First, to check if sequences from paired-end reads appear as consistent pairs in the assembly, we used Bowtie2 [27] to align the RNA-seq reads to the assembly. An average of 86% of the read-pairs, across all samples, aligned concordantly, indicating that the read pairs are well-represented in the assembly.

Next, to test the presence of contigs that are likely full-length transcripts, we used Blastx to align the contigs to two reference proteomes from UniProt. We chose the proteomes of the fruit fly (*D. melanogaster*, proteome ID: UP000000803) since it is a model organism for arthropods, and that of the whiteleg shrimp (*P. vannamei*, proteome ID: UP000283509), since it has one of the largest set of protein sequences in UniProt for crustaceans. There are 13,790 genes in the former, and 25,399 in the latter. Table 1 summarizes the alignment results. Almost 15% of both the fruit fly genes and shrimp genes are represented in almost full length (91-100%) in the assembly. Roughly 20% of both gene sets have >80% of the protein sequence locally aligned to a contig in the assembly, and this number rise to almost 30% at the >50% length coverage threshold (last row of table).

**Table 1:** Presence of almost full-length transcripts in the assembly

Perc. of protein length	No. of fruit fly proteins	No. of shrimp
aligned		proteins
91-100	2167	4015
81-100	3049	5229
71-100	3602	6118
61-100	4099	6940
51-100	4536	7794

The second (third) column shows the number of fruit fly (shrimp) proteins that align to a contig in the assembly such that the alignment covers the percentage in the first column of the protein length

Finally, the contigs were subjected to a BUSCO (version 4.0.1) analysis with the arthropoda_odb10 database, which contains 1013 BUSCO groups. We found 94.4% complete BUSCOs in the assembly, with only 4.3% missing and 1.3% fragmented. Of the complete BUSCOs, 38.3% were reported as single-copy and 56.1% duplicated, which is due to the presence of many isoforms per gene in the assembly.

### Functional annotation of de-novo assembly

**Fig. 3:**
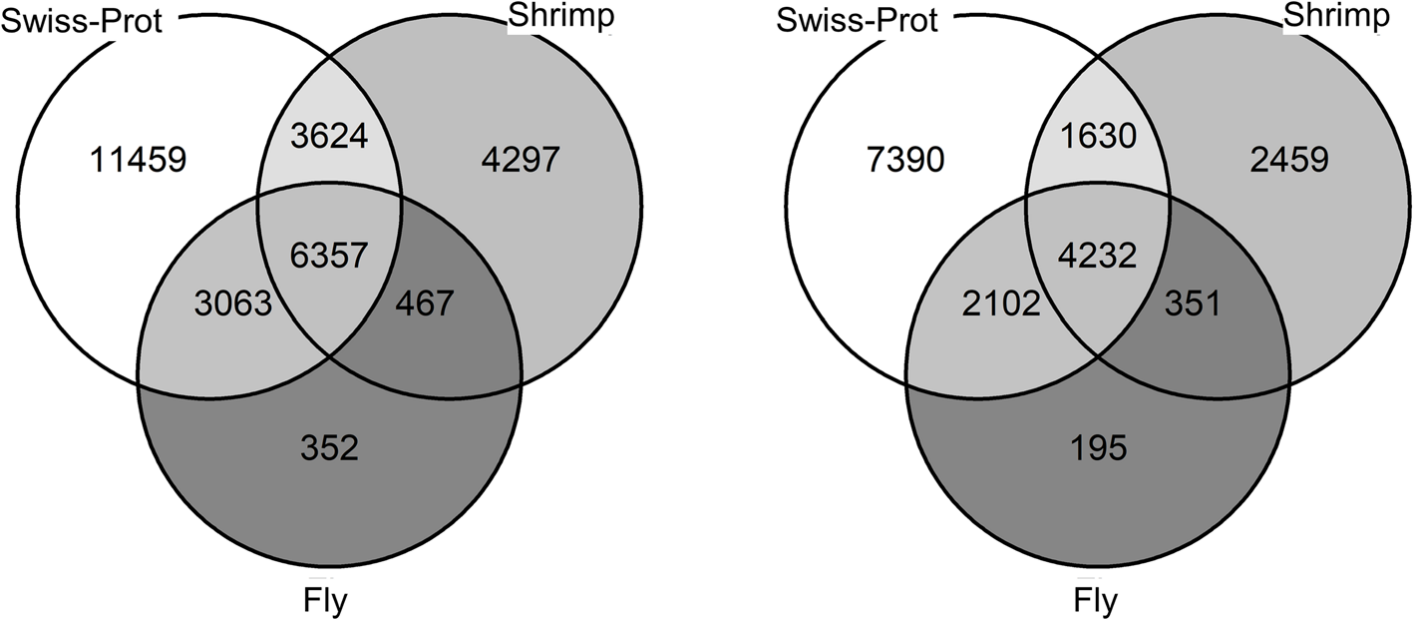
Venn diagram illustrating the number of hits found in Swiss-Prot, the fruit fly *D. melanogaster* proteome, and the shrimp *P. vannamei* proteome, for transcripts (right) and for genes (left), where a gene is said to have a hit if at least one of its isoforms in the assembly has a hit

We used the Dammit pipeline [28] which uses LAST [29] to perform a conditional reciprocal best-hit homology search [30] of our contigs against the Swiss-Prot database, the fruit fly proteome, and the proteome of the whiteleg shrimp. Of the alignments produced by Dammit, we considered as a hit only those that have an E-value at most 10^−10^ and those that cover at least 50% of the protein length. Figure 3 summarizes the results of the database hits. There were 6,356 transcripts that had a hit in each of the three databases. At the gene-level, this number reduced to 4,232.

**Fig. 4:**
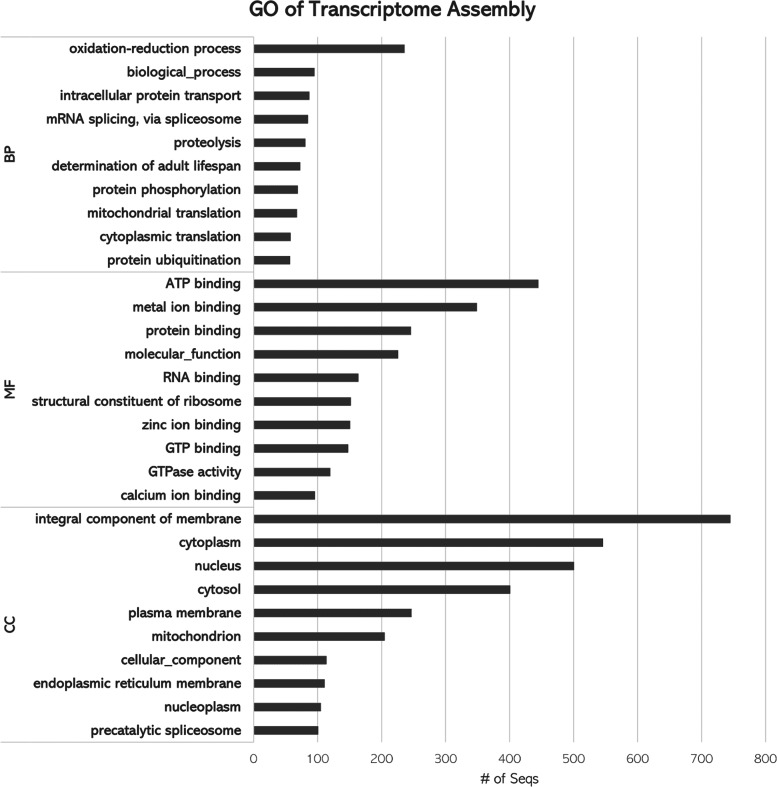
Gene ontology annotation of the de-novo transcriptome assembly. Number of transcripts assigned to top gene ontology terms for each of the three GO domains: Biological Process (BP), B) Molecular Function (MF) and C) Cellular Component (CC)

Based on the fruit fly annotations, gene ontology and pathway annotation was performed using OmicsBox and Panther [31], respectively. A total of 4,299 transcripts were assigned to one of the three GO domains: biological processes (BP), molecular function (MF) and cellular component (CC). The top gene ontology annotations (BP, MF and CC) are shown in Fig. 4. In the BP category, there was a high number of genes associated with oxidation-reduction process and protein transport and ubiquitination. These genes play a significant role in redox homeostasis, protein synthesis as well as maintaining protein structure. In the MF category, most genes were predominantly assigned to ATP binding, protein binding, RNA binding, GTP binding and calcium ion binding. These molecular activities are integral in regulation of calcium ion concentration, protein synthesis, as well as energy generation processes. In CC category, genes associated with the integral component of membrane, cytoplasm, nucleus, cytosol were found to be abundant in the transcriptome assembly.

**Fig. 5:**
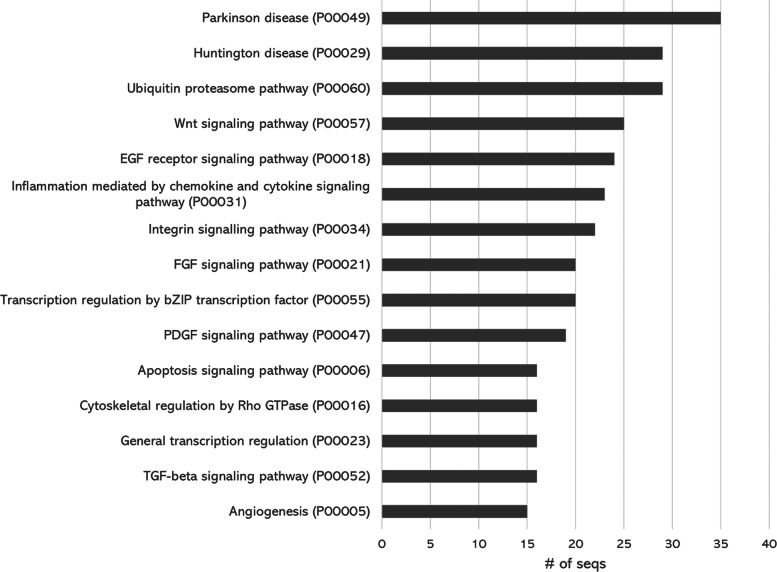
Top ten representative pathways of the de-novo transcriptome assembly annotated from the PANTHER database

Pathway analysis results are shown in Fig. 5. We found that the highest number of genes were associated with Parkinson and Huntington disease, which are model diseases for neurodegenerative disorders. Genes associated with vital signaling pathways such as Wnt signaling pathway, EGF receptor signaling pathway, Integrin signaling pathway, PDGF signaling pathway were found to be abundant in the transcriptome. We also found a high number of genes associated with ubiquitin proteasome pathway. These most-represented pathways show an overview of the cellular processes which are involved in proper biological functioning as well as the processes which may respond in reaction to heat stress.

### Differential gene expression analysis

#### Site-specific heat-stress response (main effect)

We first consider the genes which were found to be differentially expressed between the control and heat-stressed groups of a particular site. In the statistical model, these are the genes for which a site-specific main effect null hypotheses in Table 2 was rejected. We will henceforth refer to these site-specific differentially expressed genes as DE genes.

**Table 2:** For each gene, based on the model in Equation 1, we test the statistical hypotheses shown in the second column, which corresponds to the biological hypotheses in the third column

Effect type	Statistical null hypothesis	Biological null hypothesis
Main	*β*_*E*_=0	Not differentially expressed between control and heat-stressed CAG groups.
Main	*β*_*E*_+*β*_*BAT*.*E*_=0	Not differentially expressed between control and heat-stressed BAT groups.
Main	*β*_*E*_+*β*_*BIC*.*E*_=0	Not differentially expressed between control and heat-stressed BIC groups.
Interaction	*β*_*BAT*.*E*_ = 0	Effect of heat stress is not different between CAG and BAT.
Interaction	*β*_*BIC*.*E*_ = 0	Effect of heat stress is not different between CAG and BIC.

**Fig. 6:**
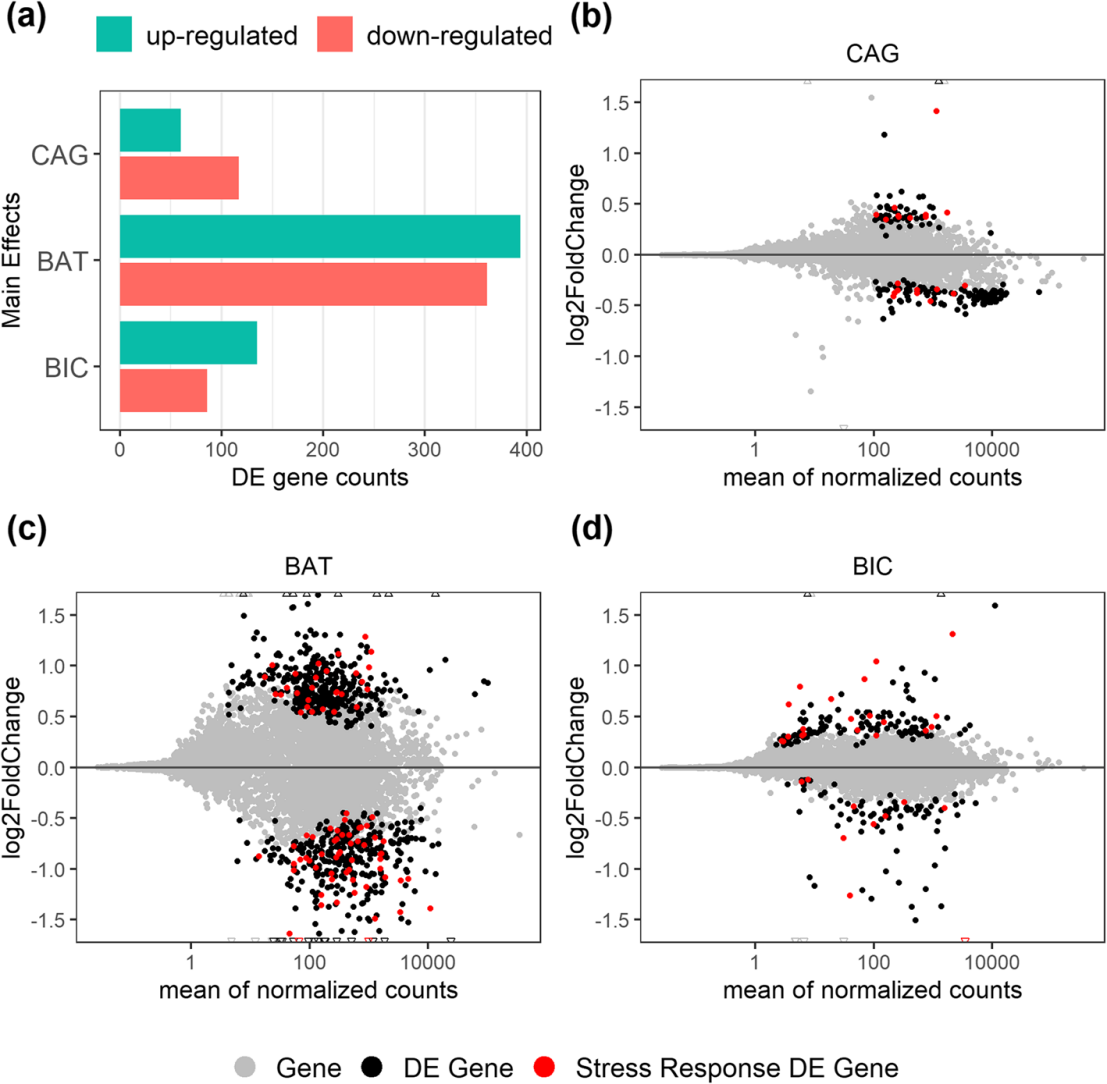
DE genes. Note: CAG = Cagayan, BAT = Bataan, BIC = Bicol (a) Bar graph showing number of DE genes classified according to the direction of regulation (b) Scatter plot of genes showing mean expression (measured as mean of normalized read counts) vs. *log*_2_-fold change for the 3 sites. Non-grey dots represent DE genes, red dots represent stress-related DE genes

There were 177, 755, and 221 DE genes in the Cagayan, Bataan, and Bicol group, respectively. As shown in Fig. 6, the number of up-regulated DE genes were 60, 394, 135 and down-regulated DE genes were 117, 361, and 86, respectively. The scatter plots in Fig. 6 show for each site, the average mean expression (as mean normalized read counts) versus the magnitude of differential expression (as *log*_2_-fold change). The DE genes are represented as non-grey dots. We call a gene to be *stress-response related* if its reciprocal-best-hit fruit fly gene is associated with a GO term that is a descendant of the GO term “response to stress” in the Gene Ontology graph. These genes can be loosely understood to be genes whose putative homologs in fruit fly have been known to be related to stress response. There were 24, 117, 39 stress-related DE genes in the Cagayan, Bataan, and Bicol group respectively, which are shown as red dots in the Fig. 6.

The list of top 20 DE genes (by adjusted *p*-value) for each site and their expression profile is shown in the Supplementary Information.

#### Across-site difference in heat-stress response (interaction effect)

We next consider the genes for which there was an effect of site in how the expression levels varied between control and heat-stressed conditions. In other words, these are genes whose differential expression was different – either in magnitude (*log*_2_-fold change) or the direction of regulation – between a site pair. In the statistical model, these are the genes for which an interaction effect null hypothesis in Table 2 was rejected. We will refer to them henceforth as differently differentially expressed (DDE) genes.

**Fig. 7:**
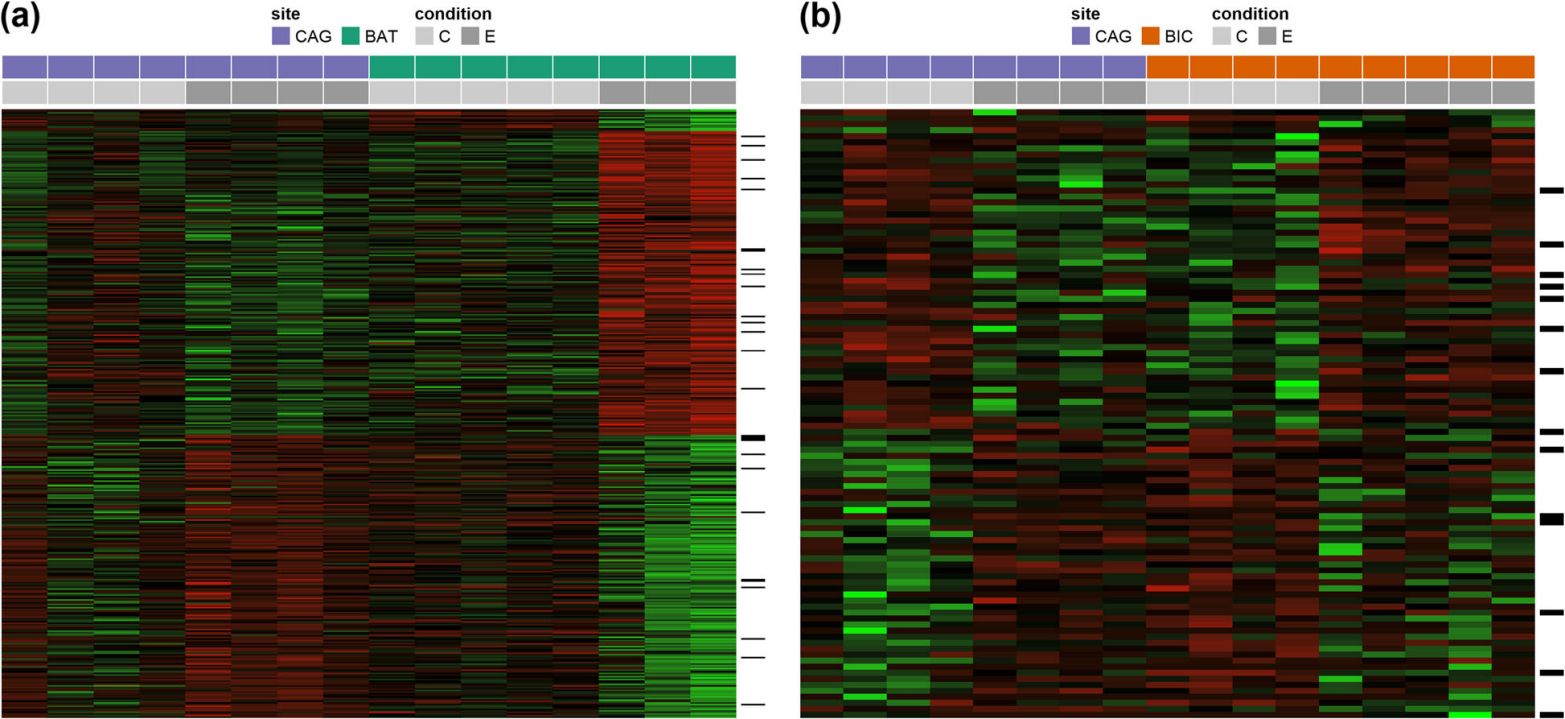
Heat map showing expression profile of the DDE genes (CAG = Cagayan, BAT = Bataan, BIC = Bicol, C = Control, E = Heat-stressed). Each row corresponds to a DDE gene, each column to a site-treatment combination, and the color of each cell represents deviation from row average of the variance-stabilized read counts – green for higher than row average, red for lower than row average, and the intensity of color represents the z-score

In the Cagayan-Bataan comparison, there were 389 DDE genes; and in the Cagayan-Bicol comparison, there were 101 DDE genes. The DDE genes can be summarized into 6 distinct categories according to the difference in direction or magnitude of the expression level variations, as shown in Table 3. The expression profiles of the DDE genes are shown as heatmaps in Fig. 7. The list of top 20 DDE genes (by size of coefficient) for the two site-pairs and the corresponding interaction plots of gene expression is provided in the Supplementary Information.

**Table 3:** Categorization of DDE genes depending on the direction or magnitude of the expression level variation between site pairs

	Uu	uU	Dd	dD	UD	DU
CAG vs. BAT	0	14	0	39	155	181
CAG vs. BIC	1	2	1	2	47	48

*Uu* more up-regulated on the first site than second, *uU* less up-regulated in the first site than second, *dD* less down-regulated in the first, *Dd* more down-regulated in the first, *UD* up-regulated in the first while down-regulated in the second, and *DU* down-regulated in the first while up-regulated in the second

GO analysis of DDE genes were performed using Blast2GO. Overall, most DDE genes were assigned to GO terms “cellular process”, “metabolic process” and “localization” in the biological process category. Next we restricted focus on the stress-related DDE genes. In the Cagayan-Bataan comparison, we observed that DDE genes associated with GO terms “activation of innate immune response”, “response to oxidative stress”, “long-chain fatty acid metabolic transport” were up-regulated in Cagayan and down-regulated in Bataan; whereas DDE genes responsible for “regulation of reactive oxygen species” and “cellular response to hypoxia” were down-regulated in Cagayan and up-regulated in Bicol. In the Cagayan-Bicol comparison, DDE genes associated with “regulation of reactive oxygen species” and “cellular response to hypoxia” were down-regulated in Cagayan and up-regulated in Bicol; whereas DDE genes associated with “response to oxidative stress”, “activation of innate immune response” and “synaptic target recognition” were up-regulated in Cagayan and down-regulated in Bicol.

Using Panther, the DDE genes were assigned to 48 and 8 reactome pathways for the Cagayan-Bataan and Cagayan-Bicol comparison, respectively. A majority of DDE genes were mapped to pathways related to stress signal transduction, metabolic processes, immune response, response to oxidative stress and cell death. When restricting focus on the stress-related DDE genes, the enriched pathways were apoptotic pathways, oxidative stress-related pathways, and signal transduction pathways such that the Integrin signaling pathway, EGF receptor signaling pathway, Interferon-gamma signaling pathway, TGF-beta signaling pathway, Wnt signaling, and p53 pathway.

## Discussion

### The first *S. serrata* transcriptome assembly

We present the first transcriptome assembly of any tissue of *S. serrata*. Several quality assessment metric attest to the reliability of the assembly. The source reads are well represented in the assembly, with an average of 86% of the read pairs aligning concordantly to the assembly. Table 1 shows that more than 4000 protein sequences of the proteome of the closely related whiteleg shrimp *P. vannamei*, align almost fully to distinct contigs in the assembly. The soundness of the assembly is further supported by BUSCO analysis, which found almost all of the 1013 arthropod BUSCOs in the assembly.

Pathway analysis results showed that our assembly is enriched in genes associated with several vital signaling pathways such as the Wnt signaling pathway, EGF receptor signaling pathway, Integrin signaling pathway, and PDGF signaling. These signaling pathways are mostly evolutionarily conserved and are responsible for proper biological functioning and development. Interestingly, these pathways are also known to function in response to heat-stress [32–35]. High number of genes associated with ubiquitin proteasome pathway may indicate activated mechanism for protein degradation, whereas apoptosis signaling pathway may indicate potential severe protein damage which leads to programmed cell death.

A few transcriptome assemblies of other *Scylla spp.* have been previously reported. These include the assemblies of the testis of *S. olivacea* [36], and testis, ovary, and infected hemocytes of *S. paramamosain*, and megalopa stage of *S. paramamosain* [37–39]. Compared to previous assemblies, our assembly was computed using the largest amount of input data so far – roughly 66 Gbases collected from 29 individuals. Functional analysis of the assembly revealed enrichment in pathways that have not been reported in any of the previous mangrove crab assemblies. This hints at a vast transcriptomic complexity in mangrove crabs; and our *S. serrata* transcriptome assembly adds to the scant but growing genomic database resources of mangrove crabs and crustaceans, in general, allowing for future investigations to shed light into the functional genomics of *Scylla spp*.

### Within-site main-effect of heat stress suggests differences in response between east and west Philippine mangrove crab populations

The number of DE genes can be indicative of the degree of modifications in cellular processes that an organism has to make in response to heat stress. In this regard, Fig. 6 shows the stark differences in how the three sites responded differently to heat stress. The remarkably high number of DE genes in the Bataan population, may be a reflection of the drastic adjustments in metabolic processes to cope with the stress or it could be a manifestation of the damaging effects of heat stress.

In contrast, the Cagayan group had a 4.5-fold lower number of DE genes and the magnitude of expression level differences, measured as log2-fold change, were also overall lower compared to Bataan. As illustrated in Fig. 1, Cagayan experiences a highly variable temperature profile. It is possible that the constant exposure to highly variable temperature prompted an increase in heat tolerance in this group which resulted in a lesser number of DE genes observed. It has been hypothesized that response to acute stress may be dependent on the magnitude of stress organisms experience throughout their lifetime, with organisms that have existed in an environment with high temperature variations having broad thermal tolerance and high capacity for acclimation [40, 41]. In addition to this, the fewer number of DE genes coupled with higher percentage of down-regulated genes observed in Cagayan population is analogous to the observations in a study done by Poong et al. [42], wherein it is considered a strategy for ‘energy and resources saving’ of heat-acclimated cells. Owing to the broad heat tolerance of the Cagayan population due to natural exposure to highly variable temperature, transcription and translation of non-essential proteins has become unnecessary which consequently saves cell energy and resources.

Interestingly, the Bicol group, whose population does not seem to have natural exposure to a wide range of temperature, demonstrated a similar response as that of Cagayan, in terms of the number of DE genes. We postulate that this similarity may be a result of population connectivity due to the established East-West separation in the Philippines. Studies on population structure of marine fishes and invertebrates in the Philippines have revealed a strong East-West separation – for example in rabbitfish [43], damselfish [44], and seahorse [45]. A study on population structure of wild *S. serrata* in the Philippines using microsatellite and mitochondrial markers also shows distinct East-West groupings: east coast populations of Cagayan and Quezon, and west coast populations of Pangasinan and Bataan [16]. For the highly dispersive *S. serrata*, the limitations on gene flow between the east and west coasts due to sea-surface currents and geographic barriers may play a significant role in shaping the adaptive genetic differences among populations in response to thermal stress. It would be interesting to disentangle the contribution of genotypic differences to divergence in expression level from that of adaptive expression changes. However there is a lack of population genomic databases for *S. serrata* which precludes such analysis.

### Analysis of interaction effect shows differences in biological pathways and genes responsible for difference in heat stress response

While the site-specific main effect analysis provides a global quantitative view of the differences in heat response, the DDE genes provide insights into the cellular processes that differentiate the heat-stress response across sites. We found that there were 48 pathways that differentially activated between Cagayan and Bataan, and 8 pathways between Cagayan and Bicol. Of particular interest are the oxidative stress and cell apoptosis pathways.

Under normal temperature conditions, the mild production of reactive oxygen species (ROS) controlled by the antioxidant defense mechanisms is helpful for proper cell function [46]. However, various stress conditions such as elevated temperature could trigger the overproduction of ROS which in turn results in cell oxidative stress [47]. Oxidative stress pathway was observed in the Cagayan-Bataan comparison with genes associated in this pathway being up-regulated in Bataan but down-regulated in Cagayan. In a study on human umbilical vein endothelial cells, it was shown that heat stress increases the ROS production and induces mitochondrial p53 translocation and Ca2+ accumulation [48]. In our study, the gene associated with the p53 pathway was up-regulated in Bataan and down-regulated in Cagayan, and genes responding to calcium ions were expressed at various levels in 3 sites. Moreover, several studies have linked ROS-induced oxidative stress to cell death in heat-exposed cells. Different pathways are responsible for cell apoptosis which include extrinsic apoptotic pathway via death receptors and intrinsic apoptotic pathway involving the mitochondria and endoplasmic reticulum [48]. Cell apoptosis pathways were observed to be differently activated in both Cagayan-Bataan and Cagayan-Bicol comparisons. Genes associated in this pathway were down-regulated in Cagayan and up-regulated in Bataan and Cagayan. Down-regulation of genes associated with oxidative stress and cell apoptosis in the Cagayan population may be attributed to the thermotolerance of the population which indicates that such changes are part of the acclimation process rather than an onset of cell death. The down-regulation of these genes may be a strategy to save energy and resources assuming that the population is already acclimated to elevated temperature.

Down-regulation of genes associated with oxidative stress and cell apoptosis in the Cagayan population may be attributed to the thermotolerance of the population which indicates that such changes are part of the acclimation process rather than an onset of cell death. The down-regulation of these genes may be a strategy to save energy and resources assuming that the population is already acclimated to elevated temperature.

### Implications for fisheries and aquaculture management of *S. serrata* populations

Aquaculture production of *S. serrata* in the Philippines reached up to 22,000 metric tons valued at Php 9 billion in 2018. As *S. serrata* is a preferred species for trading and farming, this species is subject to overexploitation for commercial purposes. Moreover, frequent exposure to environmental stressors could lead to population decline for this economically valuable crustacean. A study on domestication of mangrove crabs in the Philippines by Quinitio et al. [49] highlights the need for management of *S. serrata* populations due to a documented decrease in size, yield and catch per unit effort (CPUE) in key collection areas which are attributed to overexploitation and environmental factors.

Most mangrove crab farms in the Philippines practice a long-term grow-out approach [50] which is highly dependent on crab juveniles collected from the wild. Current hatchery and nursery production of *S. serrata* juveniles are not enough to support the needs of the mangrove crab industry in view of the species’ vulnerability to variations in environmental conditions (i.e. salinity, temperature, dissolved oxygen) especially for the early larval stages. Temperature, in particular, influences specific biological processes such as moulting, which is associated with crab growth, development, and survival [51]. Hence, knowledge on the impact of environmental conditions particularly temperature variation to the populations of *S. serrata* is critical to support their population and maintain sustainable mangrove crab aquaculture.

The findings of this study show that population-specific response of *S. serrata* to temperature variation is due to the physiological adaptation to their respective thermal optima which is derived from the local environment conditions. While our results indicate that *S. serrata* populations are capable of acclimatory plasticity, it may be metabolically expensive for the organisms to survive in a new environment with a new thermal regime. Hence in screening for potential sources of mangrove crabs for broodstock development either for aquaculture or for supportive breeding in stock management, it has been shown that one can explore the use of stocks that have broad heat tolerance and high capacity for acclimation, which may be hallmarks of success in adaptation to changing thermal environments [52].

## Conclusion

In this study, we attempted to understand the genomic and molecular mechanisms that differentiate the heat-stress response of mangrove crab populations from sites with varying climate profiles. As part of the bioinformatic analysis, we constructed a first de-novo transcriptome assembly of *S. serrata*. Several quality assessment metric point to a sound and informative assembly, which adds to the growing genomic database resources of mangrove crabs. Results from the differential expression analysis showed genes and pathways responsible for population-specific response to heat-stress in the 3 populations. Based on previous work on population structure of marine species, our findings suggest that population-specific heat-stress response might be attributed to acclimatory plasticity due to pre-exposure to extreme temperature variations or might be due to the physiological adaptation to their respective thermal optima which is derived from the local environment conditions. Our results may serve as a basis for hypothesis testing regarding the impact of population connectivity to the adaptive genetic differences among populations. Future work may focus on genetic analysis to confirm genetic signatures for thermal adaptation among populations.

## Methods

### Sample collection and thermal stress exposure

Adult *S. serrata* (90–120 mm carapace width) were collected from three sites of varying temperature profiles: Bataan, Cagayan and Bicol, Philippines. All crab samples were color-tagged according to collection sites and were subjected to formalin bath (100 ppm formalin solution) in one large tank for 1 hour. The use of formalin is a standard procedure of disinfection in aquaculture operations, in particular during quarantine when breeding stocks are first brought in from the wild or from their natural habitat [4]. A total of six groups (3 sites ×2 conditions) were considered in the experiment, with an initial count of 9 individuals per group. Prior to thermal stress exposure, all crab samples were acclimatized at an ambient temperature of 26 ±2^∘^C for 48 hours. The control condition was set at 26 ±2^∘^C for the whole duration of the study, whereas the heat stress condition was increased at a rate of 2^∘^C/24 hr until the maximum experimental temperature of 32^∘^C was reached and kept for 72 hours. The choice of temperature and duration of exposure was guided by previous stress-response studies on mud crabs. Ruscoe et al. [12] identified 30^∘^C as an ideal temperature for rearing *S. serrata*, and Yang et al. [17] indicated maximum HSP70 gene expression at 36^∘^C for 6 hours and slight elevation at 32^∘^C at 6 hours for *S. paramamosain*. We decided to expose the crabs to 32^∘^C since it is also the highest water temperature in the habitats and culture systems of *S. serrata*, and the decision for 72 hours was made from the ability of crabs to return to normal activity after exposure to 32^∘^C at 24, 48, 72, and 96 hours. All crab samples were fed with fresh squid and exposed to a salinity of 30 ±2ppt. After the heat-stress experiment, gill tissue samples were harvested, placed in RNALater (Ambion) and stored at −80^∘^C until ready for processing. Gill tissues were used based on the result of Fu et al. that expression data from hemocyte, heart, hepatopancreas and gill tissues were better than that from muscle, eyestalk, stomach, and gut tissues [53].

### RNA isolation, library construction, and RNA sequencing

Tissue samples of 30 crab samples (5 individuals from each group) were randomly selected and sent to an external service provider (Macrogen, Inc., South Korea) for RNA extraction, library construction, and sequencing. Total RNA was extracted from gill tissue following the TRIzol (Invitrogen) protocol. Quality of the extracted RNA was measured through RNA Integrity Number using 2200 TapeStation (Agilent). A total of 29 cDNA libraries (omitting one low-quality sample from Bataan-Experimental group) were constructed from poly(A)-captured mRNAs using TruSeq RNA Sample Prep Kit (Illumina, Inc.). A non-stranded RNA sequencing was performed for each library using the Illumina Hi-Seq 2000 in paired-end mode and 101 bp length.

### De-novo transcriptome assembly

SortmeRNA [54] (version 2.1b) was used to remove potential rRNA-derived reads by aligning the reads against the following databases: silva-bac-16s-id90, silva-bac-23s-id98, silva-arc-16s-id95, silva-arc-23s-id98, silva-euk-18s-id95, silva-euk-28s-id98, silva-euk-28s, rfam-5s-database-id98, rfam-5.8s-database-id98.

Trimmomatic [55] (version 0.39) was used to remove adapters and filter or trim low-quality using the following parameters: LEADING:3 TRAILING:3 AVGQUAL:20 MINLEN:35. Only those read pairs for which both members of the pair survive the filtering were kept for further analysis.

Trinity [56] (version 2.8.5), in its default settings, was applied to the entire pool of filtered reads from all samples to obtain a transcriptome assembly.

To reduce computation time of the downstream annotation pipeline, we applied CD-HIT to cluster contigs with a sequence similarity of at least 95%.

### Transcript abundance estimation

The *align_and_estimate_abundance.pl* script provided by Trinity was used to invoke the combination of RSEM [57] (version 1.3.3) and Bowtie2 [27] (version 2.3.5) to compute transcript abundance for each sample. The *abundance_estimates_to_matrix.pl* script was executed to integrate raw counts at the gene-level across samples, and also to compute cross-sample normalized TMM counts. The former was used for differential gene expression analysis, while the latter for computing ExN50 statistics and for exploratory analysis prior to differential gene expression analysis.

### Annotation and BUSCO

The Dammit annotation pipeline was used to perform conditional reciprocal best-hit search using LAST in OrthoDB, Swiss-Prot, and the UniProt reference proteomes of the fruit fly *D. melanogaster* and the whiteleg shrimp *P. vannamei*.

BUSCO analysis was performed using version 4.0.1 against the *arthropoda_odb10* database.

### Differential gene expression analysis

Differential gene expression analysis was performed on the subset of genes which had a reciprocal-best-hit alignment to a protein in the fruit fly proteome, such that the alignment had an E-value of at most 10^−10^ and it covered at least half of the protein length. There were 6,898 such genes in the assembly. While this is a conservative choice, it allows for a downstream functional analysis using the rich pathway and GO database of the fruit fly leading to more meaningful functional interpretation of the differential expressed genes. It also minimizes the possibility of contaminant contigs impacting the expression analysis.

We used DeSeq2 [58] (version 1.28.1) to perform differential gene expression analysis on the set containing Trinity-genes which found a hit in the D. melanogaster proteome with an E-value of at most 10^−10^ and the alignment covering at least 50 percent of the protein length. Transcript-level count data produced by RSEM were aggregated at the gene-level using tximport [59] (version v1.16.1). Exploratory data analysis and quality control procedures taken prior to differential gene expression analysis are described in the Supplementary Information.

For each gene, we set the DeSeq2 negative binomial regression model to a 2-factor model with interaction terms: ∼site+treatment+site:treatment, where site has 3 levels (CAG (Cagayan), BAT (Bataan), BIC (Bicol)) and treatment has 2 levels (Control (C) and Heat-stressed (E)). For the treatment factor, we chose Control as the reference level. For the site factor, we chose Cagayan as the reference level, since given its temperature extremes it faces, our prior expectation was for the Cagayan group to be the most thermally resilient. Our results suggest that this indeed seems to be the case.

With CAG and C set as reference levels for site and treatment, respectively, the regression model can be expressed as:

1 $$ \begin{aligned} \log(q_{i}) = {} &\beta_{0} + \beta_{BAT}X_{BAT} + \beta_{BIC}X_{BIC} + \beta_{E}X_{E} \\ & + \beta_{BAT.E}X_{BAT.E} + \beta_{BIC.E}X_{BIC.E}, \end{aligned}   $$

where *q*_*i*_ is the mean of negative binomial distribution for sample *i* normalized for sequencing depth, *X*s are dummy variables that take values of 0 or 1 depending on the group membership of sample *i*, and *β*s are the coefficients of the model. The hypotheses tests performed on the model coefficients are shown in Table 2

Dispersion estimates were shrunk towards the dispersion of similarly expression genes using DESeq2’s default parametric method for shrinking the dispersion. Likewise, for visualization and ranking purposes, estimated *log*_2_-fold change was shrunk towards a more likely estimate by pooling information from all genes [60]. To account for multiple testing due to the large number of genes as well as multiple comparisons per gene, we applied the Benjamini-Hochberg procedure [61] with the false discovery rate threshold at 0.1.

## Supplementary Information

**Additional file 1** Supplementary information

## Data Availability

The datasets generated during the current study are available in the DDBJ Sequence Read Archive under the accession number DRA010977. The computational pipeline used for data analysis is available at the following URL: https://bitbucket.org/s_serrata/dge/src/master/

## Abbreviations

DE: Differentially expressed
DDE: Differently differentially expressed
TMM: Trimmed mean of M values
BUSCO: Benchmarking Universal Single-Copy Orthologs
GO: Gene ontology
BP: Biological process
CC: Cellular component
MF: Molecular function
EGF: Epidermal growth factor
PDGF: Platelet-derived growth factor
ROS: Reactive oxygen species
