# Supplementary material for: Comparative transcriptome profiling of heat stress response of the mangrove crab Scylla serrata across sites of varying climate profiles
Source: BMC Genomics. 2021 Jul 29;22:580. doi: 10.1186/s12864-021-07891-w (PMC8323281; doi:10.1186/s12864-021-07891-w)
Supplement: Supplementary file 1 — Additional file 1 Supplementary information [file 12864_2021_7891_MOESM1_ESM.pdf]

Supplementary Information. Comparative transcriptome profiling of heat stress response of the mangrove crab Scylla serrata across different sites

Anish M.S. Shrestha, Crissa Ann I. Lilagan, Joyce Emlyn B. Guiao, Maria Rowena R. Romana-Eguia, and Ma. Carmen Ablan Lagman

22 November, 2020

1 Data sets

1.1 Read dataset

Table 1.1 shows the size of each RNA-seq sample.

Dataset Label = SITE_TREATMENT_SERIALNUMBER_ID
SITE is BAT for Bataan, BIC for Bicol, and CAG for Cagayan;
CONDITION/TREATMENT is C for Control, and E for Experiment (Stressed)
Filtering refers to applying sortmeRNA and Trimmomatic.

Table 1.1: Read data set

Group	No. read pairs	NO. filtered	No. mapped concordantly
BAT_C_1_39I	10,637,255	9,855,922	8,756,210
BAT_C_2_62F	11,850,257	10,966,651	9,720,512
BAT_C_3_66I	15,008,128	13,816,479	12,344,365
BAT_C_4_67M	15,417,195	14,530,199	12,905,438
BAT_C_5_68F	13,502,548	12,469,543	11,080,091
BAT_E_1_47F	16,495,146	13,644,286	12,059,468
BAT_E_2_56F	12,653,459	8,705,403	6,110,011
BAT_E_3_57I	10,924,404	9,987,890	8,915,381
BAT_E_4_65F	12,802,246	11,603,688	10,544,695
BIC_C_1_16M	12,528,914	11,730,016	10,454,352
BIC_C_2_4M	10,605,665	9,849,781	8,562,683
BIC_C_3_5F	10,356,657	9,563,229	8,235,506
BIC_C_4_64M	12,959,206	12,060,959	10,307,833
BIC_C_5_9I	12,212,734	11,367,619	9,905,726
BIC_E_1_10M	12,627,585	11,662,826	10,318,928
BIC_E_2_12I	10,686,953	10,026,910	8,735,753
BIC_E_3_26F	13,052,465	12,215,312	10,324,830
BIC_E_4_29F	10,328,697	9,624,413	8,319,072
BIC_E_5_32F	12,279,654	11,538,524	9,819,188
CAG_C_1_14F	13,698,238	12,862,738	10,920,985
CAG_C_2_41F	12,919,120	11,269,499	9,464,984
CAG_C_3_46M	10,563,472	9,810,132	8,069,261
CAG_C_4_59F	11,463,940	10,488,770	9,018,346

Group	No. read pairs	NO. filtered	No. mapped concordantly
CAG_C_5_8F	12,562,481	11,688,828	9,905,321
CAG_E_1_24I	12,396,699	11,601,679	10,181,418
CAG_E_2_28M	13,016,364	12,208,328	10,322,701
CAG_E_3_38F	15,436,337	14,450,465	12,042,491
CAG_E_4_40M	12,648,381	11,819,803	10,016,682
CAG_E_5_45F	10,855,359	10,174,839	8,467,054
Total	362,489,559	331,594,731	

1.2 Read metadata

Metadata of the samples:

Table 1.2: Read data set metadata

Sample name	Sex	Initial weight (g)	Final weight (g)	Carapice width initial(mm)	Carapice width final (mm)
BIC_E_1	M	100.8	116.4	96.58	96.66
BIC_E_2	I	88.8	97.4	91.92	92.21
BIC_E_3	F	144.0	142.7	97.69	97.77
BIC_E_4	F	147.2	147.9	98.94	98.90
BIC_E_5	F	115.6	121.1	91.67	91.92
BAT_E_3	I	221.8	236.1	117.96	117.96
BAT_E_4	F	221.5	248.3	116.44	116.44
BAT_E_1	F	243.4	258.2	115.61	115.61
BAT_E_2	F	234.3	243.4	115.30	115.30
CAG_E_2	M	192.2	159.2	100.17	100.17
CAG_E_1	I	177.6	181.4	103.85	103.85
CAG_E_3	F	159.4	167.7	100.51	100.51
CAG_E_4	M	182.7	190.4	95.91	96.76
CAG_E_5	F	163.0	166.7	103.01	103.68
BIC_C_2	M	130.1	142.3	97.20	97.23
BIC_C_1	M	135.6	142.0	93.93	93.93
BIC_C_5	I	88.3	92.3	92.20	92.33
BIC_C_4	F	107.7	119.7	93.70	93.83
BIC_C_3	F	170.3	177.5	100.54	100.55
BAT_C_1	I	197.6	212.2	112.58	112.58
BAT_C_4	M	189.6	194.4	105.43	105.43
BAT_C_2	F	245.5	252.8	114.26	114.95
BAT_C_5	F	239.8	250.9	115.43	115.43
BAT_C_3	I	206.5	219.9	108.90	108.90
CAG_C_1	F	150.8	154.9	101.04	101.94

Sample name	Sex	Initial weight (g)	Final weight (g)	Carapice width initial(mm)	Carapice width final (mm)
CAG_C_5	F	175.4	182.4	102.11	102.23
CAG_C_2	F	165.6	174.5	100.44	100.44
CAG_C_4	F	155.1	158.5	99.00	99.00
CAG_C_3	M	193.0	197.0	100.19	100.57

2 Exploratory data analysis and quality control prior to differential expression analysis

Differential expression analysis was performed on the set of genes for which we found a hit in the proteome of *D. melanogaster* such that the E-value was less than 10^{-10} and the alignment covered at least 50% of the protein length. Due to issues with data quality, we removed 4 samples, one each from the following groups BAT E, CAG C, CAG E, BIC C.

For the remaining datasets, the distribution of read counts among genes is shown in Fig. 2.1. The plots indicate that samples within each group have a similar distribution in the mid-level expression range.

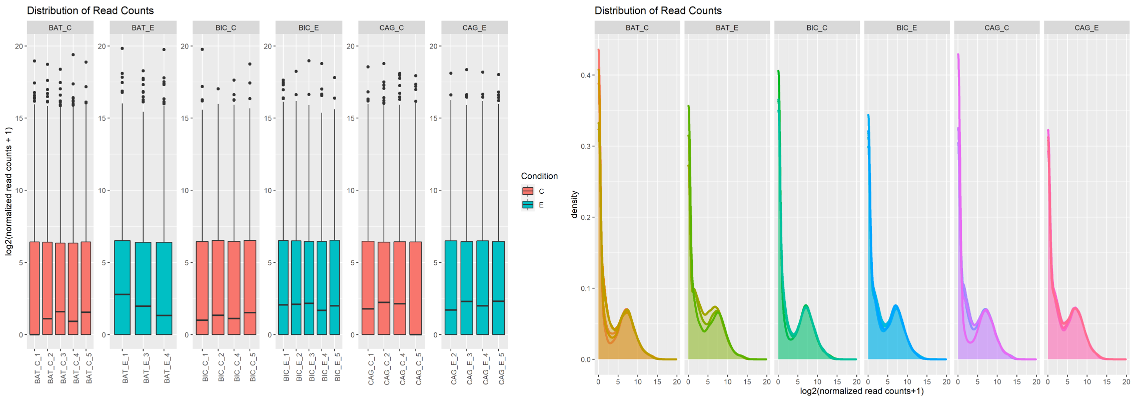

Figure 2.1: Distribution of read counts.

To further explore the within-sample similarity, we computed pairwise correlation coefficients using the count data obtained by applying a variance-stabilizing transformation to the raw counts to correct for mean-variance dependence (see Fig. 2.2). We observe high level of correlation between samples of the same group (see Fig.2.3).

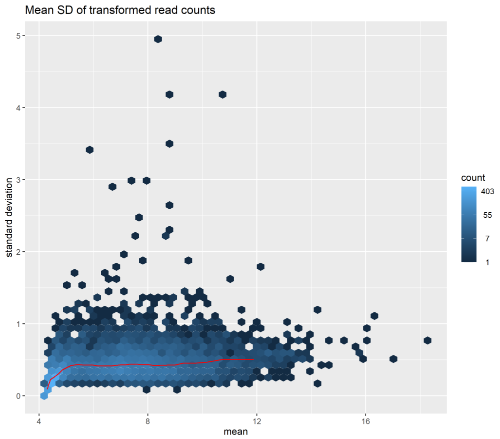

Figure 2.2: Mean-variance scatter plot after applying variance-stabilizing transformation to raw count data.

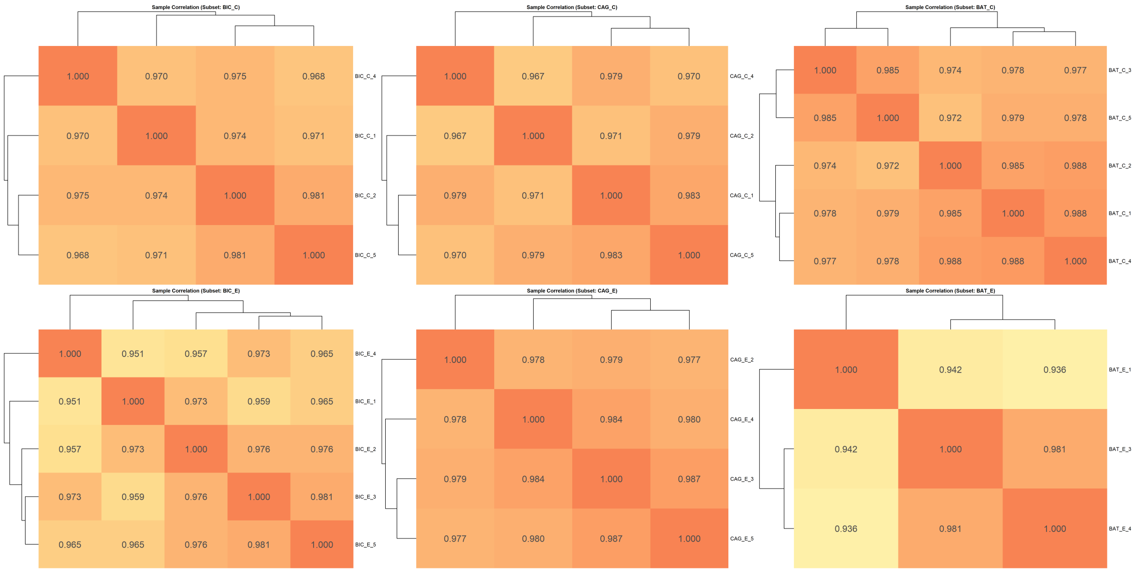

Figure 2.3: Correlation of within-group samples

Further, we performed Principal Component Analysis on the transformed data. Fig.2.4 shows the samples plotted based on the first two principal components. The first principal component seems to explain variance due to the site factor. This global view of the data provided by the PCA does not seem to clearly capture variance due to the treatment factor.

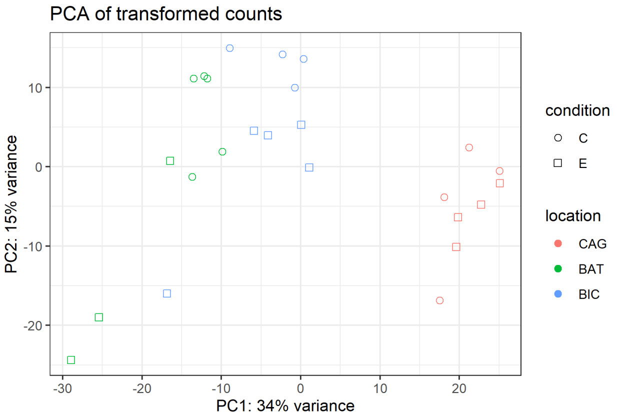

Figure 2.4: PCA clustering of the samples

Other factors, namely sex, weight, carapace width do not explain the PCA clustering, as shown in Fig.@ref(fig:pca_others) below.

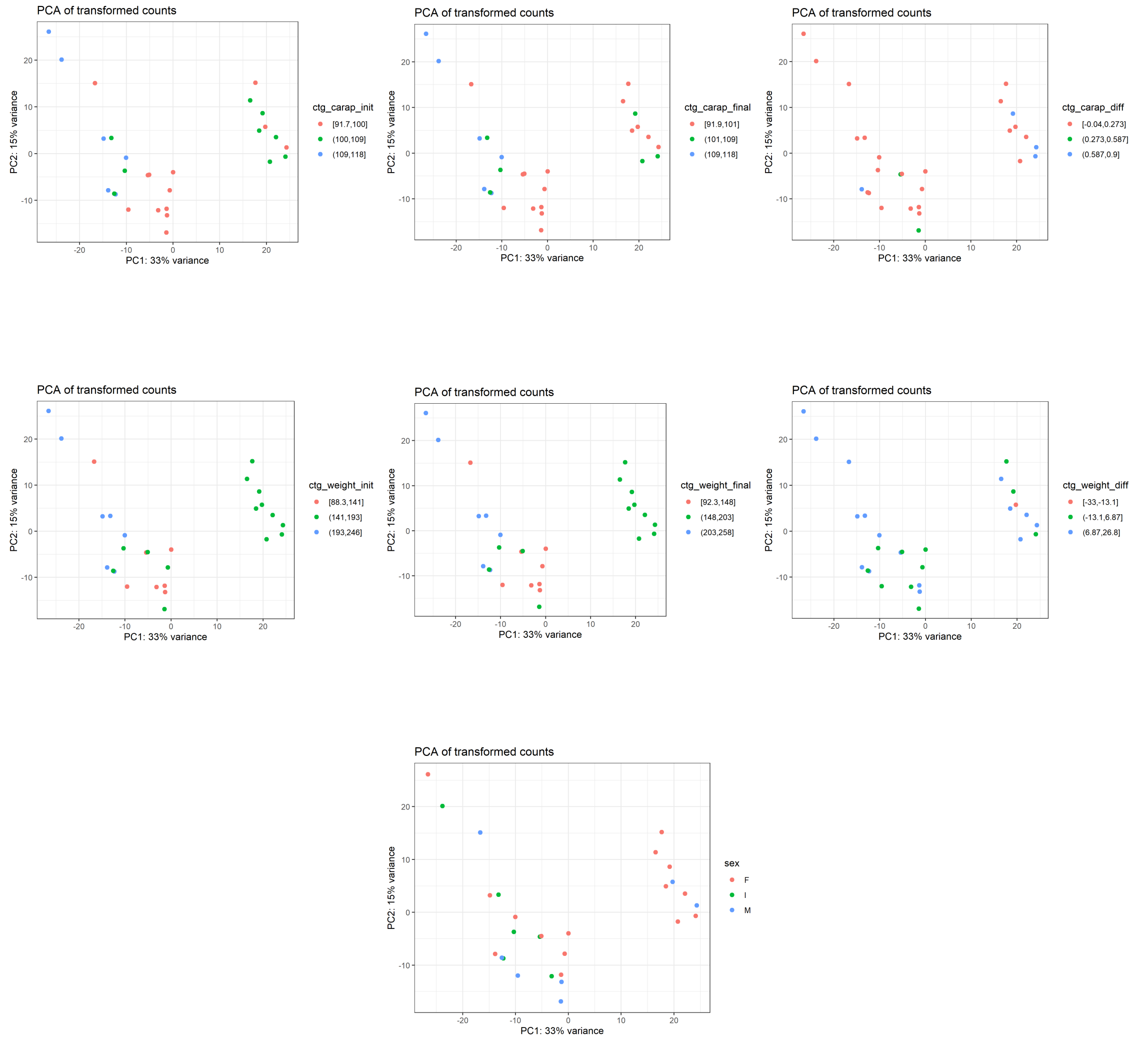

(#fig:pca_others)Checking if other factors explain the PCA

3 List of DE genes (signicant site-specific main effect of heat)

3.1 Cagayan

3.1.1 List of DE genes

(#tab:DE cag)DDE genes in Bicol-Bataan comparison

	gene_id	baseMean	log2FoldChange	lfc standard error	pvalue	p-value adjusted	Fruit fly Uniprot ID	Fruit fly Uniprot accession	Des of re G
110	TRINITY_DN4587_c0_g1	269.9982	0.2757841	0.1268240	0.0068947	0.0979492	Q9VIE7_DROME	Q9VIE7	F
136	TRINITY_DN5756_c0_g1	186.9842	-0.2908033	0.1361252	0.0068512	0.0975531	A0A0B4KHR2_DROME	A0A0B4KHR2	F
126	TRINITY_DN5216_c0_g1	158.0351	0.1921580	0.3094136	0.0068095	0.0970145	A0A0B4LFM0_DROME	A0A0B4LFM0	F
122	TRINITY_DN5061_c3_g1	169.3852	0.3446606	0.2122297	0.0065975	0.0952952	SERC_DROME	Q9VAN0	F
3	TRINITY_DN1012_c0_g1	252.8996	-0.2815411	0.2635540	0.0065523	0.0949702	Q9VJ80_DROME	Q9VJ80	F
123	TRINITY_DN5099_c0_g1	393.0791	-0.3225048	0.1580802	0.0064356	0.0937253	Q9VQ78_DROME	Q9VQ78	F

	gene_id	baseMean	log2FoldChange	lfc standard error	pvalue	p-value adjusted	Fruit fly Uniprot ID	Fruit fly Uniprot accession	Des of re G
67	TRINITY_DN2705_c2_g1	158.5210	0.3471655	0.1928971	0.0063628	0.0932941	DDB1_DROME	Q9XYZ5	F
88	TRINITY_DN3905_c0_g1	230.6792	-0.3145560	0.2463990	0.0063350	0.0931695	A1Z9J8_DROME	A1Z9J8	F
17	TRINITY_DN12245_c0_g1	686.8807	-0.3220864	0.1568217	0.0063119	0.0930174	NO66_DROME	Q7K4H4	F
97	TRINITY_DN4199_c0_g1	3450.7213	-0.2998082	0.2555043	0.0062819	0.0928246	O18680_DROME	O18680	T
42	TRINITY_DN1804_c0_g1	10431.4326	-0.3491364	0.1994885	0.0062535	0.0925681	RL13A_DROME	Q9VNE9	F
119	TRINITY_DN4957_c0_g1	358.7795	-0.3363501	0.1707080	0.0062128	0.0921832	TPPC2_DROME	Q9VUZ1	F
129	TRINITY_DN5488_c0_g1	268.8955	-0.3356495	0.1698577	0.0062127	0.0921832	Q9VN31_DROME	Q9VN31	F
84	TRINITY_DN3823_c0_g1	250.3478	-0.3494645	0.1973403	0.0061892	0.0919963	A1ZA22_DROME	A1ZA22	T
48	TRINITY_DN20560_c0_g1	148.3437	-0.3390862	0.2275231	0.0061807	0.0919250	Q7K569_DROME	Q7K569	F
164	TRINITY_DN834_c0_g1	714.6201	-0.3403143	0.1754734	0.0061711	0.0918803	Q9VEB9_DROME	Q9VEB9	T
147	TRINITY_DN6783_c0_g1	139.2259	0.2804234	0.2669614	0.0061434	0.0918058	FCL_DROME	Q9W1X8	F
38	TRINITY_DN17076_c0_g1	105.7778	0.3431134	0.2232765	0.0061046	0.0916095	Q9VUY4_DROME	Q9VUY4	F
111	TRINITY_DN46_c2_g1	558.7454	-0.3520281	0.2057795	0.0058998	0.0898429	Q9W2I4_DROME	Q9W2I4	F
16	TRINITY_DN11989_c0_g1	108.1446	-0.2643930	0.2779299	0.0058665	0.0895935	SOG_DROME	Q24025	T

3.1.2 Expression profile of the top 10 (by log-fold change) DE genes

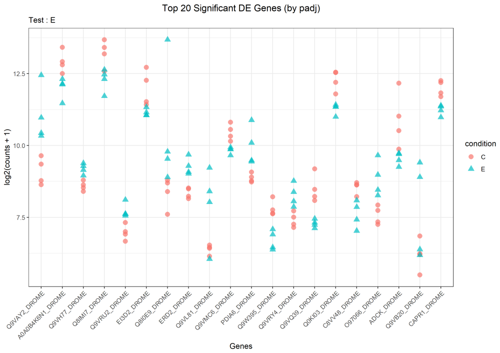

(#fig:DE cag expression)Expression profile of top 10 (by log fold change) DE genes.

3.2 Bataan

3.2.1 List of DE genes

(#tab:DE bat)DDE genes in Bicol-Bataan comparison

	gene_id	baseMean	log2FoldChange	lfc standard error	pvalue	p-value adjusted	Fruit fly Uniprot ID	Fruit fly Uniprot accession	Des of re G
298	TRINITY_DN2856_c0_g1	93.253419	0.6783260	0.3125264	0.0070856	0.0997283	Q9VHF6_DROME	Q9VHF6	F
532	TRINITY_DN5482_c0_g1	107.307434	0.7572903	0.5784951	0.0070876	0.0997283	Q9VXZ8_DROME	Q9VXZ8	F
141	TRINITY_DN16883_c0_g1	60.786052	0.5449820	0.2280520	0.0070408	0.0992934	Q9VJ11_DROME	Q9VJ11	T
480	TRINITY_DN4749_c2_g1	1686.210381	0.7464661	0.3772898	0.0070071	0.0990404	Q9VV75_DROME	Q9VV75	F
598	TRINITY_DN6625_c0_g3	321.807206	0.6630777	0.3000147	0.0069781	0.0986861	RM19_DROME	Q9VHN6	F
32	TRINITY_DN1117_c1_g2	5278.686452	-0.6974971	0.3270314	0.0069728	0.0986666	Q9VB22_DROME	Q9VB22	F

	gene_id	baseMean	log2FoldChange	lfc standard error	pvalue	p-value adjusted	Fruit fly Uniprot ID	Fruit fly Uniprot accession	Des of re G
194	TRINITY_DN2023_c0_g1	361.550855	0.6209288	0.2714086	0.0069689	0.0986666	Q7JRE1_DROME	Q7JRE1	F
5	TRINITY_DN10207_c0_g1	26.414808	0.7205609	0.3476890	0.0069384	0.0982920	DPOE3_DROME	Q9V444	[illegible]
669	TRINITY_DN8204_c0_g1	88.646012	-0.7502488	0.3781673	0.0067917	0.0969809	Q9V9S9_DROME	Q9V9S9	F
29	TRINITY_DN11123_c0_g1	122.363379	0.5735513	0.2420264	0.0067624	0.0967494	CMTR2_DROME	Q9UAS6	F
26	TRINITY_DN11005_c0_g1	4083.691404	0.7025492	0.3284824	0.0067171	0.0962449	Q9VIQ8_DROME	Q9VIQ8	F
454	TRINITY_DN44819_c0_g1	74.495227	0.7323161	0.3560706	0.0066882	0.0961263	Q9VL22_DROME	Q9VL22	F
489	TRINITY_DN4834_c0_g1	1228.193318	-0.6375464	0.2797072	0.0066529	0.0958743	A0A0B4JD64_DROME	A0A0B4JD64	[illegible]
269	TRINITY_DN2594_c0_g1	1595.083357	0.6278066	0.2733867	0.0066460	0.0958418	RENR_DROME	Q9VHG4	F
270	TRINITY_DN2601_c0_g1	1081.569437	0.4111909	0.1621032	0.0066014	0.0952962	MED14_DROME	Q9W0P8	F
739	TRINITY_DN9671_c0_g2	4.338600	0.7403976	0.6124380	0.0065691	0.0951041	A0A0B4LFL3_DROME	A0A0B4LFL3	F
239	TRINITY_DN23513_c0_g2	23.576204	-0.7956813	0.4643865	0.0065611	0.0950424	Q9Y1A7_DROME	Q9Y1A7	F
291	TRINITY_DN28016_c0_g3	6.302175	0.7807571	0.5605081	0.0065307	0.0948768	E1JJM9_DROME	E1JJM9	F
200	TRINITY_DN2069_c0_g1	111.931858	0.6662655	0.2971258	0.0064057	0.0935495	Q9VL89_DROME	Q9VL89	F
589	TRINITY_DN6402_c0_g4	330.909763	0.7560288	0.3777741	0.0063838	0.0934334	Q9W127_DROME	Q9W127	F

3.2.2 Expression profile of the top 10 (by log-fold change) DE genes

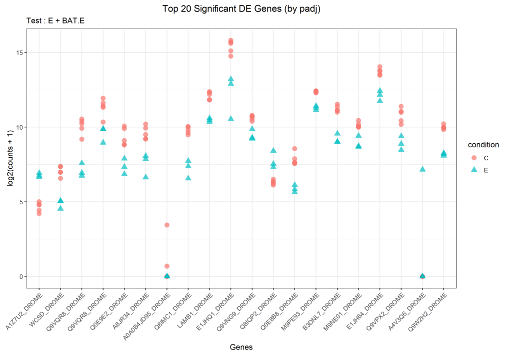

(#fig:DE bat expression)Expression profile of top 10 (by log fold change) DE genes.

3.3 Bicol

3.3.1 List of DE genes

(#tab:DE bic)DDE genes in Bicol-Bataan comparison

	gene_id	baseMean	log2FoldChange	lfc standard error	pvalue	p-value adjusted	Fruit fly Uniprot ID	Fruit fly Uniprot accession	Descenda of Stress reponse GO term
19	TRINITY_DN123_c3_g1	63.637472	-0.3348664	0.2382543	0.0070605	0.0995141	M9PG83_DROME	M9PG83	TRUE
71	TRINITY_DN23088_c0_g1	6.011408	-0.1243049	0.5522271	0.0070321	0.0992260	M9PCC1_DROME	M9PCC1	FALSE
147	TRINITY_DN4656_c0_g1	109.360280	0.3149391	0.1880752	0.0070222	0.0991425	O97183_DROME	O97183	FALSE
21	TRINITY_DN12613_c0_g1	224.799715	-0.3435357	0.2662161	0.0068856	0.0978762	M9PCQ1_DROME	M9PCQ1	FALSE
74	TRINITY_DN2352_c0_g1	321.995220	-0.3439709	0.2644768	0.0068047	0.0970145	Q7KSF5_DROME	Q7KSF5	TRUE
157	TRINITY_DN53907_c0_g1	12.438436	0.3626503	0.4911122	0.0068086	0.0970145	O62526_DROME	O62526	FALSE

	gene_id	baseMean	log2FoldChange	lfc standard error	pvalue	p-value adjusted	Fruit fly Uniprot ID	Fruit fly Uniprot accession	Descenda of Stress reponse GO term
207	TRINITY_DN8199_c0_g1	6.459856	0.2948902	0.5404713	0.0067677	0.0967494	A1Z8U9_DROME	A1Z8U9	FALSE
136	TRINITY_DN43617_c0_g1	6.775130	-0.1225245	0.5814979	0.0067402	0.0965204	SAHH2_DROME	Q9VZX9	FALSE
10	TRINITY_DN1117_c1_g2	5278.686452	-0.3529194	0.3029661	0.0067142	0.0962449	Q9VB22_DROME	Q9VB22	FALSE
33	TRINITY_DN1488_c0_g1	2305.728268	-0.2610372	0.1275686	0.0066896	0.0961263	M9PEX2_DROME	M9PEX2	FALSE
70	TRINITY_DN23007_c0_g1	6.521468	0.3219995	0.5434182	0.0065892	0.0952811	Q9VFV9_DROME	Q9VFV9	TRUE
128	TRINITY_DN4054_c1_g1	3110.957327	-0.3337521	0.2205130	0.0065450	0.0949198	Q9VN29_DROME	Q9VN29	FALSE
214	TRINITY_DN9256_c0_g1	114.512823	0.3726568	0.4128577	0.0064580	0.0939838	COQ6_DROME	Q9VMQ5	FALSE
134	TRINITY_DN42781_c0_g1	12.691116	-0.2698686	0.5388643	0.0064264	0.0936879	Q8IRZ1_DROME	Q8IRZ1	FALSE
138	TRINITY_DN43963_c0_g1	7.689503	-0.1207304	0.6310230	0.0064167	0.0936015	Q9VAY2_DROME	Q9VAY2	TRUE
212	TRINITY_DN9083_c0_g1	477.167423	0.3744378	0.4737619	0.0064107	0.0935684	E1JIU2_DROME	E1JIU2	FALSE
155	TRINITY_DN5282_c6_g1	125.782851	-0.3528670	0.2828318	0.0063696	0.0932941	Q9VGM4_DROME	Q9VGM4	FALSE
217	TRINITY_DN9393_c0_g2	60.347884	-0.3763530	0.4499996	0.0063616	0.0932941	A1Z7H2_DROME	A1Z7H2	FALSE
161	TRINITY_DN5533_c0_g1	1105.651122	-0.3305317	0.2077475	0.0063227	0.0930418	Q7KVT8_DROME	Q7KVT8	FALSE
44	TRINITY_DN16356_c0_g1	31.157241	0.3757328	0.4067427	0.0062451	0.0924987	Q8SXK2_DROME	Q8SXK2	FALSE

3.3.2 Expression profile of the top 10 (by log-fold change) DE genes

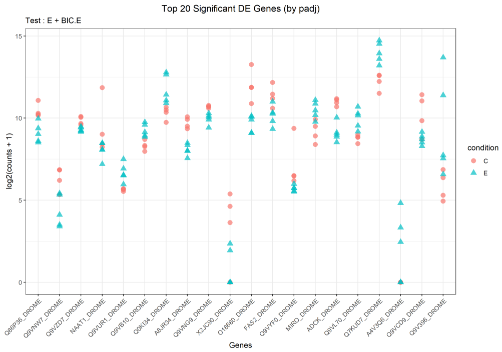

(#fig:DE bic expression)Expression profile of top 10 (by log fold change) DE genes.

4 List of DDE genes

4.1 Cagayan-Bataan Comparison

4.1.1 Gene List

(#tab:DDE cag_v_bat)DDE genes in Cagayan-Bataan comparison

	gene_id	baseMean	log2FoldChange	lfc standard error	pvalue	p-value adjusted	Fruit fly Uniprot ID	Fruit fly Uniprot accession	Desce of S repo GO
126	TRINITY_DN23186_c0_g1	32.47371	-18.154820	3.9187482	1.00e-07	0.0000897	A0A0B4JD95_DROME	A0A0B4JD95	TR
189	TRINITY_DN3316_c0_g1	65.80276	-5.651525	1.6659434	0.00e+00	0.0000291	H8F4T3_DROME	H8F4T3	FAI
74	TRINITY_DN1700_c0_g1	980.59320	-2.654224	0.5355785	0.00e+00	0.0000000	Q9VQR8_DROME	Q9VQR8	FAI
127	TRINITY_DN2351_c0_g1	1163.88215	-2.459218	0.5812316	0.00e+00	0.0000003	Q8IMC1_DROME	Q8IMC1	FAI

	gene_id	baseMean	log2FoldChange	lfc standard error	pvalue	p-value adjusted	Fruit fly Uniprot ID	Fruit fly Uniprot accession	Desc of S rep GO
364	TRINITY_DN872_c1_g1	1031.26994	-2.092963	0.5001875	0.00e+00	0.0000006	Q9W2H2_DROME	Q9W2H2	FA
8	TRINITY_DN10839_c0_g1	34.36582	-2.083861	0.9742235	9.00e-07	0.0003470	Q9VGN6_DROME	Q9VGN6	FA
71	TRINITY_DN1684_c0_g1	174.32938	-1.563603	0.4880310	7.00e-07	0.0003013	WCSD_DROME	Q9VXV9	FA
90	TRINITY_DN19044_c0_g1	57.32281	-1.488798	0.7697045	1.56e-05	0.0024694	A0A0B4JD63_DROME	A0A0B4JD63	FA
75	TRINITY_DN1700_c0_g2	1256.83442	-1.482509	0.4218800	6.00e-07	0.0002543	Q9VQR8_DROME	Q9VQR8	TR
353	TRINITY_DN823_c7_g1	119.21309	-1.471902	0.5902991	7.60e-06	0.0015635	Q9VTS0_DROME	Q9VTS0	FA
233	TRINITY_DN43139_c0_g2	60.49644	-1.374946	0.5846743	1.94e-05	0.0028877	Q95SM8_DROME	Q95SM8	FA
359	TRINITY_DN8394_c0_g1	177.82714	-1.329096	0.7162333	4.32e-05	0.0051041	Q9VNG6_DROME	Q9VNG6	TR
236	TRINITY_DN4417_c0_g1	143.01379	-1.282654	0.4459534	1.75e-05	0.0026399	Q0E8B8_DROME	Q0E8B8	FA
345	TRINITY_DN7875_c0_g1	1495.36528	-1.270153	0.3923570	8.30e-06	0.0016539	Q9VPX2_DROME	Q9VPX2	FA
12	TRINITY_DN1116_c0_g1	347.28020	-1.260356	0.5268485	4.60e-05	0.0052802	MY31D_DROME	Q23978	TR
346	TRINITY_DN7889_c0_g1	278.44889	-1.244530	0.4292108	2.31e-05	0.0031782	Q7K1L4_DROME	Q7K1L4	FA
109	TRINITY_DN20978_c0_g1	506.68924	-1.231685	0.3850188	1.25e-05	0.0022067	A8JR34_DROME	A8JR34	FA
263	TRINITY_DN5064_c0_g1	29.17502	-1.216071	0.4456262	4.10e-05	0.0049662	DONS_DROME	Q9VNA8	FA
290	TRINITY_DN6_c0_g1	155.12707	-1.213972	0.6787232	9.54e-05	0.0081548	A0A0B4K6A6_DROME	A0A0B4K6A6	TR
356	TRINITY_DN8299_c0_g1	124.32612	-1.197957	0.5152706	8.37e-05	0.0074876	Q9VWJ7_DROME	Q9VWJ7	FA

4.1.2 Interaction plots for DDE genes between Cagayan and Bataan

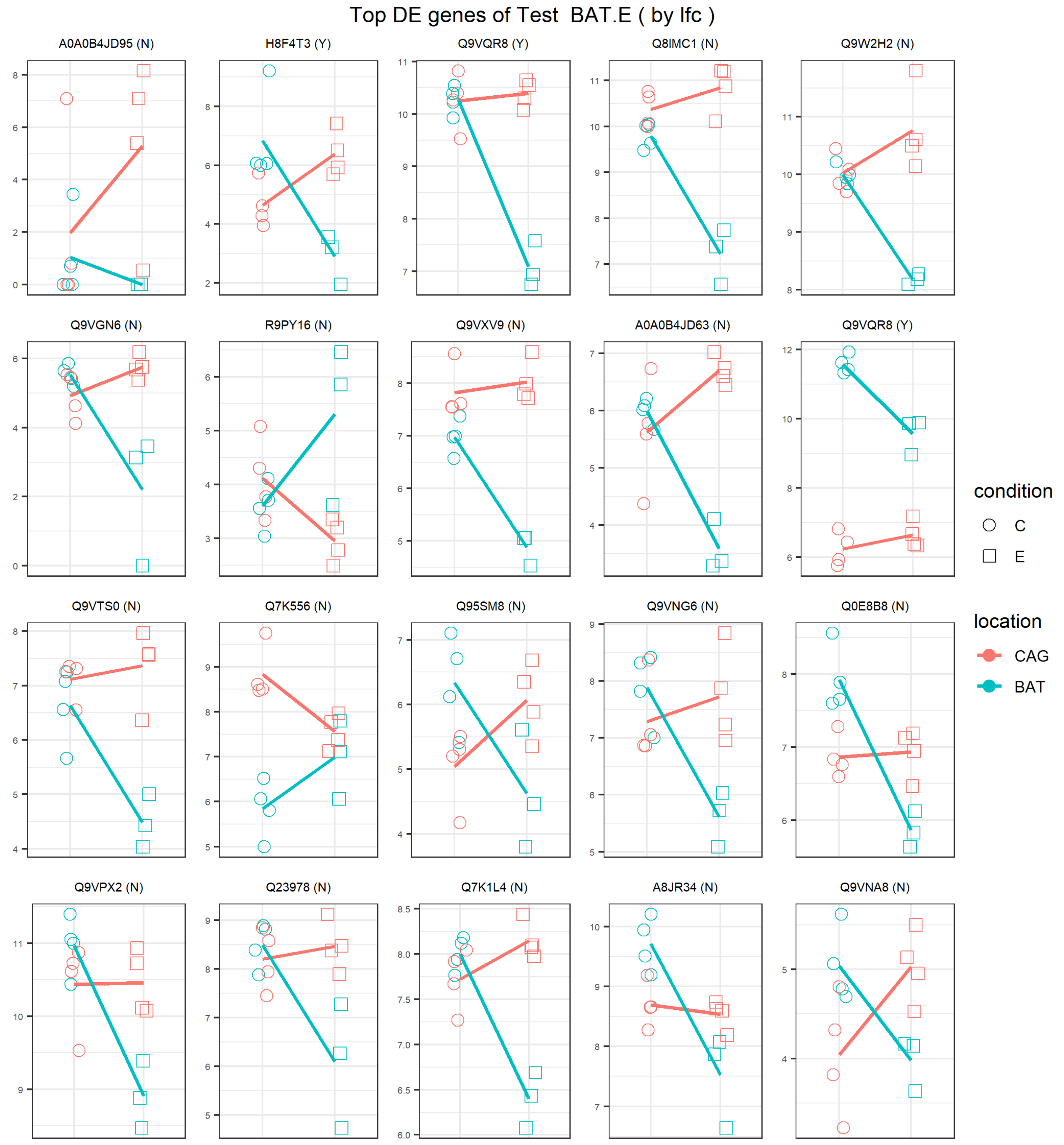

(#fig:interaction CAG BAT)Interaction plots for Cagayan-Bataan comparisons

4.2 Cagayan-Bicol Comparison

4.2.1 Gene list

(#tab:DDE cag_v_bic)DDE genes in Cagayan-Bicol comparison

	gene_id	baseMean	log2FoldChange	lfc standard error	pvalue	p-value adjusted	Fruit fly Uniprot ID	Fruit fly Uniprot accession	Desc of S rep GO
50	TRINITY_DN3107_c0_g2	737.41943	-0.3887049	0.1749122	0.0006752	0.0268445	A0A0B4KEU5_DROME	A0A0B4KEU5	FA
73	TRINITY_DN5212_c1_g1	67.86881	-0.3685687	0.1969325	0.0004867	0.0226173	OFUT2_DROME	Q9W589	FA
32	TRINITY_DN20978_c0_g1	506.68924	-0.3615601	0.2079321	0.0003083	0.0173752	A8JR34_DROME	A8JR34	FA
45	TRINITY_DN2870_c0_g1	649.95675	-0.3513920	0.1883893	0.0013400	0.0398601	M9PC47_DROME	M9PC47	FA

	gene_id	baseMean	log2FoldChange	lfc standard error	pvalue	p-value adjusted	Fruit fly Uniprot ID	Fruit fly Uniprot accession	Desc of S rep GO
34	TRINITY_DN2351_c0_g1	1163.88215	-0.3472561	0.2159184	0.0002990	0.0170070	Q8IMC1_DROME	Q8IMC1	FA
41	TRINITY_DN260_c0_g1	280.88154	-0.3411983	0.2144442	0.0003667	0.0188634	Q9VZ56_DROME	Q9VZ56	FA
70	TRINITY_DN4699_c0_g1	128.85973	-0.3351064	0.1791266	0.0030263	0.0644374	Q9XZ19_DROME	Q9XZ19	FA
54	TRINITY_DN32760_c0_g1	155.41135	-0.3282156	0.1972950	0.0015910	0.0439830	A0A0B4LHX1_DROME	A0A0B4LHX1	FA
8	TRINITY_DN13010_c0_g1	68.46256	-0.3167548	0.1946409	0.0026628	0.0601383	Q7JRD4_DROME	Q7JRD4	FA
49	TRINITY_DN3052_c1_g1	150.69126	-0.3157591	0.2571398	0.0002112	0.0136705	Q9VL81_DROME	Q9VL81	FA
71	TRINITY_DN4928_c0_g1	1274.48494	-0.3108055	0.1255183	0.0031292	0.0657493	RPB1_DROME	P04052	FA
4	TRINITY_DN11231_c0_g1	160.65039	-0.3072568	0.4332258	0.0001133	0.0092104	NOCT_DROME	A8JQX3	FA
48	TRINITY_DN30180_c0_g1	100.00087	-0.2983928	0.1992533	0.0033405	0.0676907	Q0KHU2_DROME	Q0KHU2	FA
28	TRINITY_DN19301_c0_g1	38.44343	-0.2981740	0.1984424	0.0035649	0.0694561	LIMK1_DROME	Q8IR79	FA
93	TRINITY_DN799_c0_g1	354.85133	-0.2964378	0.1998585	0.0033816	0.0679191	PCAT_DROME	Q0KHU5	FA
3	TRINITY_DN1111_c0_g1	285.91173	-0.2938369	0.1904903	0.0063903	0.0934334	XYLK_DROME	Q95T10	FA
9	TRINITY_DN13170_c0_g1	28.89774	-0.2902338	0.2575152	0.0003245	0.0177454	SY65_DROME	P21521	FA
36	TRINITY_DN2430_c2_g1	36.14537	-0.2889838	0.2047434	0.0028226	0.0620669	A0A0B4KH03_DROME	A0A0B4KH03	FA
61	TRINITY_DN35089_c0_g1	46.27318	-0.2849461	0.1980169	0.0053807	0.0855536	Q9VZD9_DROME	Q9VZD9	FA
66	TRINITY_DN4036_c0_g1	90.16868	-0.2834029	0.3874633	0.0001698	0.0119170	D2MP_DROME	Q8MPP3	TI

4.2.2 Interaction plots

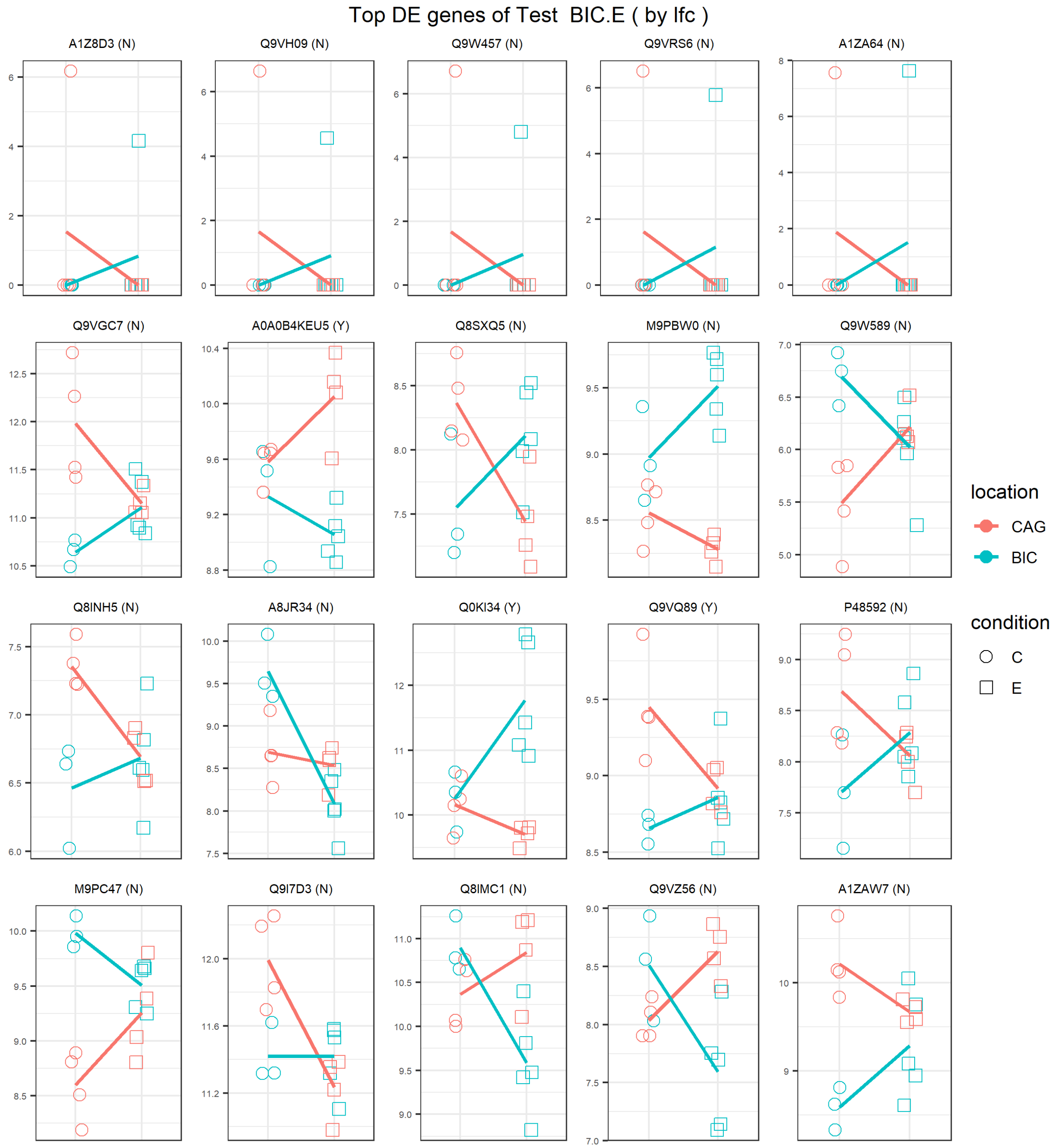

(#fig:interaction CAG BIC)Interaction plots for Cagayan-Bataan comparisons
